# Acute pontine infarction after percutaneous coronary intervention: a very rare but devastating complication

**DOI:** 10.1007/s12471-015-0717-2

**Published:** 2015-06-03

**Authors:** F. Arslan, J. Mair, W.-M. Franz, M. Otten, L. van Lelyveld

**Affiliations:** 1 Department of Cardiology, University Medical Center Utrecht, Heidelberglaan 100, 3584 CX Utrecht, The Netherlands; 2 Department of Internal Medicine, Diakonessenhuis Utrecht, Utrecht, The Netherlands; 3 Department of Internal Medicine III—Cardiology and Angiology, Innsbruck Medical University, Innsbruck, Austria; 4 Department of Intensive Care Medicine, Diakonessenhuis Utrecht, Utrecht, The Netherlands

**Keywords:** Embolic stroke, Percutaneous coronary intervention, Acute myocardial infarction

## Abstract

A 64-year-old man suffering from an acute posterior wall myocardial infarction underwent primary percutaneous coronary intervention. After several aspiration attempts, tirofiban infusion and pre- and post-dilatation, a bare-metal stent was successfully implanted in the culprit right coronary artery. While the patient did not show any neurological symptoms before or during the procedure, he exhibited hemiplegia and loss of spontaneous speech. Additional magnetic resonance imaging showed an extensive brain stem infarction. This is the first report of a brain stem infarction as a complication of percutaneous coronary intervention.

**Electronic supplementary material:**

The online version of this article (doi:10.1007/s12471-015-0717-2) contains supplementary material, which is available to authorized users.

Here we present a case of a 64-year-old man, without any history of cardiovascular risk factors or other comorbidities, who underwent acute percutaneous coronary intervention (PCI) for an acute posterior wall myocardial infarction. Coronary angiography revealed an occluded right coronary artery (Fig. 1a, Online Video 1). In addition, the left circumflex coronary artery showed a non-occlusive thrombus, and the left anterior descending coronary artery revealed a Thrombolysis in Myocardial Infarction grade II flow (Fig. 1b, Online Video 2). After several aspiration attempts and intracoronary administration of tirofiban, the right coronary artery could be reopened with several pre-dilatations and implantation of a Driver Sprint bare-metal 4.5 15-mm stent (Medtronic Inc, Minnesota, USA) with subsequent 5.0 NC balloon post-dilatation. Because of the high thrombus load, successful reperfusion of the posterolateral branch was not possible (Fig. 1c, Online Video 3). A temporary pacemaker was placed before the PCI due to bradycardia. After the procedure, the patient became hemiplegic and exhibited loss of spontaneous speech. Cerebral magnetic resonance imaging revealed an extensive left-sided infarction in the brain stem expanding into the cerebral and cerebellar peduncle (Fig. 2). Thrombolysis was not possible, as dual anti-platelet therapy, heparinisation and continuous intravenous tirofiban had already been initiated. Subsequently, he was mechanically ventilated and treated with antibiotics for 6 days after which he developed aspiration pneumonia caused by neural dysphagia. Although he was extubated after 9 days, he received a tracheostoma due to persistent dysphagia and high risk for aspiration. He was transferred to the stroke unit for further rehabilitation. Coronary angiography and/or percutaneous coronary interventions have been associated with cerebral ischaemic complications. The complication rate varies between 0.07 and 0.4 % of the procedures [1–4]. However, a brain stem infarction is highly unusual compared with cerebral stroke considering the difficult anatomical path for an embolus originating from the aortic arch. Before the embolus reaches the basilar artery, it needs to pass through the subclavian (or the brachiocephalic trunk for right-sided lesions) and the vertebral artery. To our knowledge, this is the first report of an acute pontine infarction as a complication of percutaneous coronary intervention.

**Fig. 1:**
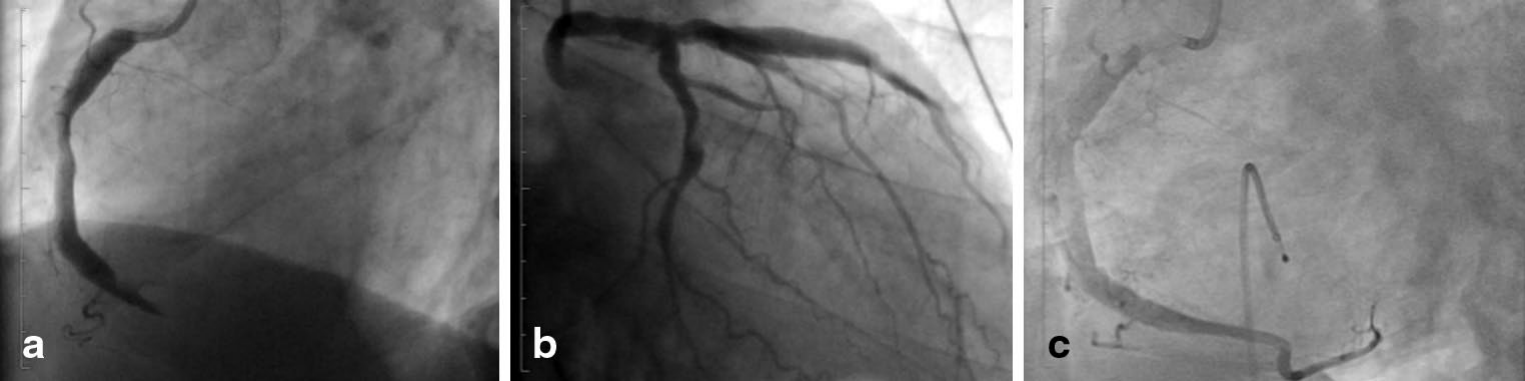
Coronary angiography of the **a** culprit lesion in the right coronary artery, **b** stenotic lesion in the left circumflex coronary artery and **c** right coronary artery after stent placement

**Fig. 2:**
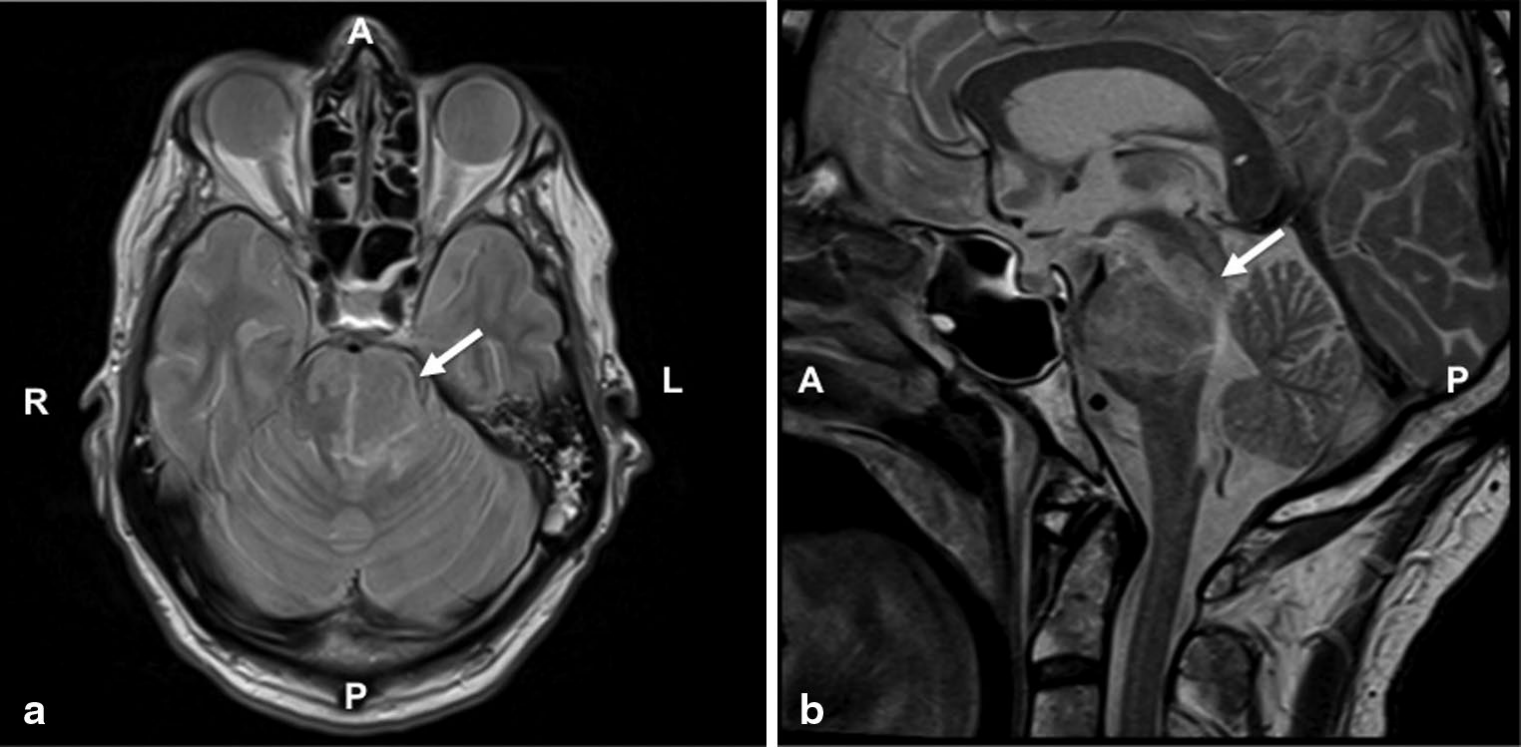
Transversal and sagittal views of the brain stem using T2-weighted magnetic resonance imaging. A left-sided brain stem infarction expands to the cerebral and cerebellar peduncle (*arrows*). *A* anterior, *P* posterior, *R* right, *L* left

## Online video legends

**Online Video 1(MOV 612 kb)** Coronary angiography showing a totally occluded right coronary artery

**Online Video 2(MOV 424 kb)** Coronary angiography showing Thrombolysis in Myocardial Infarction grade II flow in the left anterior descending coronary artery and a non-occlusive thrombus in the left circumflex coronary artery

**Online Video 3(MOV 899 kb)** Coronary angiography showing Thrombolysis in Myocardial Infarction grade III flow in the right coronary artery after percutaneous coronary intervention with stent placement
